# Role of Macrophage Colony-Stimulating Factor for Staphylococcal Infection in the Oral Cavity

**DOI:** 10.3390/jcm12185825

**Published:** 2023-09-07

**Authors:** Hidenobu Senpuku, Kazuhisa Yoshimura, Hideki Takai, Yutaka Maruoka, Erika Yamashita, Akira Tominaga, Yorimasa Ogata

**Affiliations:** 1 Department of Bacteriology I, National Institute of Infectious Diseases, Tokyo 162-8640, Japan; 2 Department of Microbiology and Immunology, Nihon University of School of Dentistry at Matsudo, Matsudo 271-8587, Japan; 3 Tokyo Metropolitan Institute of Public Health, Tokyo 169-0073, Japan; kazuhisa_yoshimura@member.metro.tokyo.jp; 4 Department of Periodontology, Nihon University School of Dentistry at Matsudo, Matsudo 271-8587, Japan; takai.hideki@nihon-u.ac.jp (H.T.);; 5 National Center for Global Health and Medicine, Tokyo 162-8655, Japan; ymaruoka@hosp.ncgm.go.jp; 6 Department of Orthodontics, Nihon University School of Dentistry at Matsudo, Matsudo 271-8587, Japan; maer20024@g.nihon-u.ac.jp

**Keywords:** M-CSF, CA-125/MUC15, staphylococci, saliva, elderly, HIV

## Abstract

Objective: There are few valid indicators of oral infection owing to the complexity of pathogenic factors in oral diseases. Salivary markers are very useful for scrutinizing the symptoms of disease. To provide a reliable and useful predictive indicator of infection for opportunistic pathogens in individuals with compromised immune systems, such as those with periodontal diseases and Human Immunodeficiency Virus (HIV), this study examines opportunistic pathogens such as *C. albicans* and staphylococci and macrophage colony-stimulating factor (M-CSF) and CA125/MUC16 in saliva. The aim was to explore the correlations investigated among these factors. Methods: Samples were divided into two groups (based on patient sex, the absence and presence of dentures in elderly, or HIV-positive patients and healthy subjects), and the correlation was analyzed in two groups of elderly patients with periodontal disease (64.5 ± 11.2 years old) and HIV-infected patients (41.9 ± 8.4 years old). Healthy subjects (33.8 ± 9.1 years old) were also analyzed as a control. Levels of *C. albicans*, staphylococci, and M-CSF, which is an immunological factor for the differentiation of macrophage, and CA125/MUC16, which provides a protective lubricating barrier against infection, were investigated. Results: A significant and positive correlation between the levels of M-CSF and staphylococci was found in elderly individuals and HIV-positive patients treated with antiretroviral therapy. A significant and positive correlation between the levels of M-CSF and CD125/MUC16 was also found in both patients. These correlations were enhanced in both patients as compared with healthy subjects. Conclusion: Salivary M-CSF might be useful as a new indicator of opportunistic infection caused by staphylococci and a defense against infection in immunocompromised hosts.

## 1. Introduction

Indicators of infection with opportunistic pathogens such as *Staphylococcus aureus* and *Candida albicans* are important for determining the local and systemic risk of diseases and planning treatment for prevention. However, there are few valid indicators of infection, as it is difficult to identify indicators owing to complex pathogenic factors in the oral cavity. Aspiration pneumonia has also attracted attention as a disease of the elderly population involving infection with microorganisms from the oral cavity. Among commensal oral bacteria, pneumonia-related bacteria include *Streptococcus pneumoniae*, *Klebsiella pneumoniae*, and *S. aureus* [1,2]. These bacteria are rarely detected in the oral cavity of healthy and young people, but the quantity of these bacteria increases when elderly individuals are bedridden, or their immunity is compromised due to systemic infectious diseases such as HIV [1].

Evidence suggests that *S. aureus*, *Klebsiella oxytoca*, *S. pneumoniae*, and *C. albicans* might be associated with opportunistic infections in the oral cavity and the development of aspiration pneumoniae in elderly individuals [3]. *S. aureus* has been found more commonly in those infected with HIV [3]. Many investigators have indicated that the oral cavity functions as a potential reservoir for *S. aureus* infection in immunosuppressed patients [4,5] and it has also been recognized as an etiological factor of infective endocarditis [6]. *S. aureus* is more frequently detected in elderly individuals with periodontal diseases than in healthy subjects [7]. Additionally, *S. aureus* has been reported to cause systemic diseases such as chronic kidney disease, orofacial granulomatosis, and Crohn’s disease [8,9,10]. The mechanism associated with opportunistic infection in the oral cavity is not well understood. In addition, the oral cavity factors that support infection with opportunistic bacteria have not yet been clarified.

Macrophage colony-stimulating factor (M-CSF) is an essential growth factor that controls the proliferation, differentiation, and survival of hematopoietic lineages, including monoblasts, promonocytes, monocytes, macrophages, and osteoclasts [11,12,13,14]. M-CSF is constitutively expressed in most tissues and various types of cells, including monocytes, fibroblasts, osteoblasts, stromal cells, endothelial cells, and tumor cells, and is readily detected in circulation. In mice, the levels of granulocyte macrophage colony-stimulating factor (GM-CSF) and M-CSF in the lung are low but increase during inflammation, and these cytokines may have a role in macrophage polarization [15]. M-CSF but not GM-CSF exhibits increased serum levels in symptomatic coronavirus disease 2019 (COVID-19) patients compared with asymptomatic or convalescent patients [16]. Furthermore, M-CSF is associated with the differentiation of monocytes into HIV-resistant macrophages promoted by IL-27 [17]. Therefore, M-CSF-differentiated macrophages are susceptible to viral infection and correlate with higher mortality due to viral disease [18]. These protective γδ T cells exhibit a distinct transcriptional profile that includes abundantly expressed M-CSF, which protects against *Plasmodium* recurrence [19]. Lung-resident natural Th17 cells and γδ T cells control opportunistic *S. aureus* infection [20]. Therefore, elevated M-CSF levels in serum may mirror the infection status of various pathogens. MUC16, also known as CA125, is thought to provide a protective lubricating barrier against infectious agents and particles on mucosal surfaces [21,22]; thus, CA125/MUC16 may represent a marker for infection. Saliva is a useful sample for diagnosing infections in the oral cavity. Targeting M-CSF and CA125/MUC16 in saliva samples from elderly patients with periodontal disease and HIV-positive patients has shown encouraging results, supporting previous reports investigating the use of these markers for assessing opportunistic infection.

Elderly individuals often suffer from periodontal disease, and HIV-positive patients are often treated with ART to control the viral load. Elderly and HIV-positive patients are immunocompromised hosts susceptible to opportunistic infection. In this study, to provide a reliable and useful clinical diagnostic and predictive indicator for opportunistic infection in patients, such as elderly patients with periodontal diseases and HIV-positive patients treated with ART, various parameters were measured, and correlations were statistically analyzed. This research identified a new indicator for predicting opportunistic infections. This indicator may be useful for maintaining oral and general health in immunocompromised hosts such as elderly and HIV-positive patients.

## 2. Materials and Methods

### 2.1. Study Samples

Elderly patients with periodontal diseases were randomly selected among ambulatory patients on the basis of age 60 or older. In this cross-sectional study, we enrolled 30 individuals (total: 64.5 ± 11.2 years old; female: 64.5 ± 14.0 years old; male: 64.6 ± 9.5 years old) from Nihon University School of Dentistry at Matsudo (Matsudo, Japan) who had periodontal disease, were orally examined, and agreed to provide a saliva sample (Table 1). We do not have precise information on oral care in house. Periodontal examination and saliva sampling were performed before scaling. Therefore, professional care did not directly affect results. Periodontal examination and saliva sampling were performed before scaling. HIV-positive patients (male) were divided into ART(+) patients who had a positive response to ART and ART(−) patients who did not have a positive response. HIV-positive patients (total numbers (n = 40): 41.9 ± 8.4; ART (+, 31): 41.8 ± 8.4 years old; ART (−, 9): 42.1 ± 8.8 years old) were randomly selected among ambulatory patients from the National Center for Global Health and Medicine (Tokyo, Japan), National Hospital Organization Osaka National Hospital (Osaka, Japan), and National Hospital Organization Nagoya Medical Center (Nagoya, Japan). Medical histories included 5 with diabetes, 3 with hepatitis C, 3 with syphilis, 1 with hepatitis B, and 1 with Kaposi’s sarcoma in HIV-positive patients. However, even if they were taking mediation, this study did not factor in the symptoms or presence of these diseases when evaluating its results. As a control, healthy male subjects (n = 28, 33.8 ± 11.2 years old) who did not have periodontal disease were randomly selected among residents from the National Center for Global Health and Medicine (Tokyo, Japan) and National Institute of Infectious Disease (Tokyo, Japan) for comparison with HIV patients. The study protocol was approved by the Research Ethics Committees of the National Institute of Infectious Diseases and Nihon University of Dental School at Matsudo, Japan (No. 580 and 683), and all participants provided written informed consent. All methods were performed in accordance with the relevant guidelines and regulations. Ages, sex differences, and the presence or absence of dentures may have affected the oral flora. Participants were asked to answer a questionnaire regarding oral health and data concerning age, sex, presence of diseases, and use of dentures. Therefore, elderly patients were grouped based on male or female and denture use or no denture use.

### 2.2. Clinical Status Examination

Clinical parameters were recorded by a single inspector blinded to the type of treatment the subject received. The clinical parameters of the subjects were recorded by a periodontologist who was approved by the Japanese Society of Periodontology and trained in periodontology at Nihon University Dental Hospital at Matsudo. The numbers of teeth were counted in patients. Patients with few teeth used partial mobile prostheses. The clinical parameters plaque control record (PCR), tooth mobility (TM), probing pocket depth (PPD), clinical attachment level (CAL), and bleeding on probing (BOP) were measured for each subject. PCR was used to monitor oral hygiene during the course of the study [23]. TM was recorded based on the criteria described by Ramford et al. [24,25]. The PPD was recorded from the gingival margin to the bottom of the periodontal pocket using a periodontal probe with 1-mm markings. Measurements were made at 6 sites per tooth, and if the measurements fell between markings, the closest millimeter measurement was recorded. The CAL was measured in millimeters from the cementoenamel joint to the end of the pocket. Following PPD measurements, bleeding was determined by the presence or absence of BOP at 6 sites per tooth. BOP was recorded as “0” or “1” if no bleeding occurred or if bleeding occurred within 10 s after probing, respectively. Improvement in the CAL was considered the primary endpoint. The secondary endpoints were improvement in PPD, BOP, TM, and PCR.

### 2.3. Saliva Collection

Stimulated saliva was collected in a conical tube while chewing 0.5 g of paraffin for 5 min. The sample was immediately frozen at −20 °C and sent to the National Institute of Infectious Diseases, Department of Bacteriology I (Tokyo, Japan). After thawing frozen saliva, the saliva sample was centrifuged to remove debris, and the supernatant was subdivided and used for M-CSF and CA125/MUC16 analyses.

### 2.4. Quantification of Colony Numbers of Microorganisms in the Oral Cavity

In the direct sampling approach, a sterile cotton swab (Eiken Chemical Co., Ltd., Tokyo, Japan) was lightly rubbed against the buccal mucosa 6 times, and 2 rubbed swabs were inserted into the primary isolation medium and sent to the National Institute of Infectious Diseases, Department of Bacteriology I (Tokyo, Japan). One rubbed swab was added to 2 mL of sterile PBS, the bacteria were lightly dispersed by ultrasonic waves, and then the bacterial suspension was diluted and inoculated on *Staphylococcus*-selective medium (mannitol salt agar plate, Nissui Pharmaceutical Co., Ltd., Tokyo, Japan) to assess staphylococci and CHROMagar *Candida* (CHROMagar, Paris, France) to assess *Candida* spp. *Staphylococcus* and *Candida* were cultured under aerobic conditions for 48 h at 37 °C.

### 2.5. M-CSF and CA125/MUC16 Immunoassay

Concentrations of M-CSF and CA125/MUC16 in saliva were quantified using a commercially available enzyme-linked immunosorbent assay (ELISA) according to the manufacturer’s instructions (R & D Systems, Minneapolis, MN, USA). The sample was thawed and centrifuged at 10,000× *g* rpm for 5 min before analysis. The detection limit for quantification was 78.1 pg/mL, and the assay sensitivity was 11.2 pg/mL. A microplate spectrophotometer (SpectraMAX 340, Sunnyvale, CA, USA) was used to read the absorbance at 450 nm after the reaction, and the wavelength correction set to 540 nm was subtracted. The total amounts of M-CSF and CA125/MUC16 were determined by the Bradford assay to allow normalization of M-CSF and CA125/MUC16 levels based on total salivary protein.

### 2.6. Statistical Analysis

Data analyses were performed using Statistical Package for Social Sciences (SPSS) version 20 (IBM Corporation, Armonk, NY, USA). Continuous variables are presented as the mean and standard deviation (SD), and categorical variables are presented as frequencies. The results from 2 groups were analyzed using an unpaired Student’s *t*-test. Correlations among parameters were determined by the Pearson correlation coefficient. Statistical significance was set at *p* < 0.05.

## 3. Results

The status of periodontal disease in elderly individuals was checked. The clinical parameters in periodontal disease, levels of *S. aureus* and *C. albicans*, and concentration of M-CSF in saliva are presented in Table 1. Data were divided into two groups (based on patient sex and absence or presence of dentures) and compared for elderly patients with periodontal disease. Regarding periodontal disease parameters, elderly patients without dentures showed significantly greater PPD than those with dentures. There were no significant differences in other parameters between patients with dentures and patients without dentures or between males and females. There were no significant differences in the levels of staphylococci and *C. albicans* between females and males or patients with dentures and without dentures. Regarding M-CSF and CA125/MUC16, there were no significant differences in any of the comparisons.

Table 2 summarizes the combinations of parameters for which a significant correlation was found. In periodontal patients, PPD was significantly correlated with TM, PCR, and BOP. Furthermore, TM was significantly correlated with BOP and CAL. CAL was significantly correlated with BOP. Although these were expected results, the parameters of periodontal diseases were associated with each other. The level of M-CSF was significantly and positively correlated with the level of staphylococci and the concentration of CA125/MUC16 in saliva from elderly individuals. The number of teeth was significantly and negatively correlated with the level of *C. albicans*.

In the female group, the correlations with these parameters were analyzed (Supplementary Figure S1). Significant and positive correlations were found between PD and BOD, CAL, and TM. There were no significant correlations between *C. albicans* and other parameters. A significant correlation between M-CSF and staphylococci was found. In another observation, correlations were found between elderly patients without dentures, and these correlations were analyzed within the parameters of periodontal disease (Supplementary Figure S1). Significant and positive correlations were found between PPD and BOP, PCR, or TM; BOP and PCR or TM; CAL and TM or BOP. A correlation between M-CSF and staphylococci was also found in the two groups. Elderly individuals with periodontal disease (male) but not dentures were omitted from the analysis of correlation because the number of subjects was small (≤10). Taken together, the periodontal disease status was confirmed in elderly individuals.

The differences among healthy subjects, all HIV-positive patients, and ART(+) and ART(−) HIV-positive patients were investigated based on saliva samples. Levels of CD4 were normal, but levels of *C. albicans* were significantly and positively higher in HIV-positive ART(+) patients than in healthy subjects (Table 3). The level of *C. albicans* was more than 19 and 476 times higher in ART(−) than in ART(+) and healthy subjects, respectively, but there was no significant difference. There were no significant differences in other comparisons between parameters. Correlations among parameters were analyzed for HIV-positive patients and healthy subjects. A significant correlation between the levels of M-CSF and staphylococci was found among all HIV-positive patients and ART(+) patients (Table 4). Furthermore, a significant correlation between the levels of M-CSF and CA125/MUC16 was also found among all HIV-positive patients and ART(+) patients but not among healthy subjects. The correlation between M-CSF and staphylococci or CA125/MUC16 was stronger in HIV-positive ART(+) patients than in healthy subjects.

Significant correlations between M-CSF and streptococci were recognized in all groups (Table 2, Table S1 and Table 4); however, the correlation was strongest in HIV-positive ART(+) patients, and the correlation between M-CSF and CA125/MUC16 was higher in HIV-positive ART(+) patients and elderly individuals than in healthy subjects (Table 2 and Table 4).

## 4. Discussion

M-CSF is widely distributed in multiple species, including mammals, birds, and teleosts; it is an important cytokine with pleiotropic roles in the proliferation, survival, and differentiation of macrophages and their respective progenitors and participates extensively in the antimicrobial immune response [26,27,28]. Dreschers et al. speculated that increased M-CSF levels might reflect the process of inflammation that is associated with microbial infection [29]. M-CSF levels correlated significantly with the level of staphylococci in elderly individuals with periodontal disease and HIV-positive patients. This correlation was also observed in healthy subjects. Therefore, M-CSF seems to be specifically associated with opportunistic infection with staphylococci in humans without disease conditions. However, the correlation was noted in hosts such as elderly and HIV-positive patients.

Numerous studies have demonstrated the effects of M-CSF on macrophage activation, maturation, and inflammatory responses, such as macrophage priming, cytokine production, leukocyte recruitment, the acquired response, and the pathogenic infection response [30]. Since M-CSF is involved in antimicrobial immune activities, its expression has been considered to be induced by pathogenic infection. It was reported that the M-CSF level was dramatically increased upon challenge with lipopolysaccharide, a component of gram-negative bacteria [31], or with infectious agents such as *C. albicans* [32] and *Listeria monocytogenes* [33]. M-CSF expression in macrophages is also induced by infection with *Mycobacterium marinum* and *Leishmania major* [34,35]. M-CSF is a central regulator of macrophage function and can promote the activation of macrophage defense mechanisms against pathogens [36,37]. However, the elderly had periodontal diseases, which are infectious diseases caused by oral microorganisms, but M-CSF did not correlate with various parameters in the development of periodontal diseases. Therefore, M-CSF may be specifically associated with staphylococcal infection in the oral cavity rather than the onset and progression of periodontal disease.

ART quickly suppresses HIV-1 replication and controls HIV-1 infection. ART in the chronic phase of HIV-1 infection decreases but maintains T-cell activation. Early ART during acute HIV-1 infection rapidly reduces the levels of immune activation markers, but the rate of decline of T-cell activation is not faster [38,39,40,41,42]. HIV infection and ART administration might impact the balance of the oral microbiome, and dysbiosis of the oral microbiome can be partially restored after ART. However, these changes in the oral microbiome of HIV patients may reflect the immune status of patients. Opportunistic infection is common among HIV-infected patients even in the era of ART [43,44]. Reports have indicated that ART administration can affect the balance of the salivary microbiome in HIV-infected patients. In this study, the level of *C. albicans* was reflected in the change in the salivary microbiome in ART(+) patients. Susceptibility to opportunistic infection is enhanced by some weakened immune activities caused by HIV infection, even in patients treated with ART. However, the level of *C. albicans* was not correlated with the concentration of M-CSF and CD4^+^ cells. HIV infection and ART administration are likely to respond to the relationship between the levels of salivary M-CSF and staphylococci more than *C. albicans.* This may indicate the complexity of mucosal immunity in the salivary microbiome restored by ART.

MUC16, which is a transmembrane mucin and a high-molecular-weight glycoprotein, may be cleaved on the cell surface infected by staphylococci [45,46]. MUC16 is presented and released on the apical surface of the epithelial membrane, and the formation of a glycan-enriched barrier by MUC16 protects against pathogen contact. A previous report presented an increase in the adherence of *S. aureus* after MUC16 knockdown [47] and indicated the protective responses of MUC16 to staphylococcal infection. In this study, a positive correlation between M-CSF and CD125/MUC16 was observed in elderly individuals with periodontal disease and HIV-positive patients. Positive correlations were also observed in the healthy control group and similar to data in another group [48]. However, the degree of the correlation was not particularly strong. The correlation between the M-CSF and MUC16 levels was likely to develop in HIV-positive patients compared with healthy controls.

The number of teeth was significantly and negatively correlated with the amount of *Candida* in periodontal disease patients. Fewer teeth are associated with the use of dentures, and the attachment of those dentures may be associated with *Candida* infection in the oral cavity. *Candida* is a commensal microbe in the oral cavity of 46–65% of healthy individuals, and in denture wearers, the prevalence of *Candida* increases to 60–100% [49,50,51]. Therefore, the number of teeth might be indirectly associated with *Candida* infection.

M-CSF in saliva may be a robust indicator of opportunistic infection by staphylococci and defense against the infection in the oral cavity. These results may provide new indicators for the diagnosis of opportunistic infections accompanied by oral dysbiosis that become a problem for immunocompromised hosts such as elderly patients with periodontal disease and HIV-positive patients. Screening for salivary M-CSF is easily performed by ELISA in a few hours. Furthermore, an M-CSF detection kit using immunochromatography can also be useful in the medical office. Therefore, knowledge of M-CSF levels may be useful for maintaining oral and general health. However, there were limitations in this study related to sample size, variation in active salivary factors, other opportunistic pathogens, and regional differences in patient demographics in this study. Future large-scale surveys of various factors, including other cytokines, in patients with various diseases are required.

## 5. Conclusions

M-CSF, for screening opportunistic pathogens in the oral cavity of elderly patients with periodontitis and HIV-positive patients, was identified as a potential indicator of staphylococcal infection and a protective lubricating barrier for the infection. The correlation between M-CSF and staphylococci or CA125/MUC16 was enhanced in these patients compared with healthy subjects. M-CSF might be a risk indicator for opportunistic infection by staphylococci in the oral cavity.

## Figures and Tables

**Table 1:** Parameters between male and female, and denture (+) and denture (−) in elderly.

Indicators	Elderly Patients				
	Total (30)	Male (9)	Female (21)	Denture (+, 10)	Denture (−, 20)
Age	64.5 ± 11.2	61.3 ± 17.3	64.5 ± 12.0	66.3 ± 7.2	63.6 ± 12.8
Numbers of teeth	22.1 ± 5.3	21.9 ± 6.6	22.2 ± 4.8	16.1 ± 3.5	25.2 ± 2.9
PPD	2.94 ± 0.53	3.02 ± 0.81	2.91 ± 0.37	2.73 ± 0.16	3.05 ± 0.61
PCR	23.7 ± 16.6	17.6 ± 18.2	26.3 ± 15.6	20.8 ± 18.2	25.2 ± 16.1
BOP	12.0 ± 15.0	13.2 ± 20.5	11.5 ± 12.5	11.4 ± 12.6	12.3 ± 16.3
TM	0.27 ± 0.39	0.29 ± 0.51	0.26 ± 0.34	0.23 ± 0.29	0.29 ± 0.43
CAL	4.06 ± 1.03	4.37 ± 1.36	3.93 ± 0.86	3.66 ± 0.86	4.27 ± 1.09
Staphylococci	122.2 ± 316.3	57.0 ± 99.9	150.2 ± 371.9	91.3 ± 133.7	137.8 ± 378.7
*C. albicans*	147.1 ± 466.8	8.4 ± 17.7	206.6 ± 550.9	304.5 ± 763.0	68.5 ± 193.1
M-CSF	1285.2 ± 1102.5	846.9 ± 390.2	1473.5 ± 1256.4	1331.4 ± 1220.6	1262.1 ± 1071.6
CA125/MUC16	297.6 ± 252.5	205.1 ± 145.5	217.3 ± 146.1	195.9 ± 150.3	179.9 ± 135.0

**Table 2:** Correlations among parameters in elderly patients with periodontal diseases.

Indicators	Elderly Patients	
	Correlation	*p*-Value
PPD vs. TM	0.696	0.000 **
PPD vs. PCR	0.371	0.044 *
PPD vs. BOP	0.751	0.000 **
BOP vs. TM	0.409	0.025 *
CAL vs. TM	0.667	<0.001 **
CAL vs. BOP	0.393	0.032 *
M-CSF vs. Staphylococci	0.469	0.009 **
M-CSF vs. CA125/MUC16	0.615	0.003 **
Numbers of teeth vs. *C. albicans*	−0.398	0.029

*: *p* < 0.05, **: *p* < 0.01.

**Table 3:** Parameters in HIV patients.

Indicators	Healthy Subjects (28)	HIV Patients		
		Total (40)	ART+ (31)	ART− (9)
Age	33.8 ± 9.1	41.9 ± 8.4	41.8 ± 8.4	42.1 ± 8.8
Staphylococci	302.6 ± 1140.6	743.4 ± 2325.0	582.2 ± 1829.4	1298.6 ± 3654.1
*C. albicans*	4.32 ± 14.1	546.3 ± 2188.5	107.5 ± 183.7 *	2057.4 ± 4461.8
CD4^+^ cells		413.0 ± 245.1	512.0 ± 200.3	225.9 ± 233.9
M-CSF	862.8 ± 620.5	1315.0 ± 1079.5	1374.0 ± 1188.2	1111.9 ± 571.0
CA125/MUC16	297.6 ± 252.5	325.4 ± 343.9	334.6 ± 371.2	293.3 ± 241.8

*: *p* < 0.01. vs. healthy subjects.

**Table 4:** Correlations among parameters in Healthy subjects and HIV-positive patients.

	Healthy Subjects		HIV Patients (Total)		HIV Patients (ART+)	
	Correlation	*p*-Value	Correlation	*p*-Value	Correlation	*p*-Value
M-CSF vs. Staphylococci	0.429	<0.029 *	0.296	<0.027 *	0.582	<0.001 **
M-CSF vs. CA125/MUC16	0.370	0.063	0.602	<0.001 **	0.575	<0.001 **

*: *p* < 0.05, **: *p* < 0.01.

## Data Availability

All data generated or analyzed during this study are included in this published article.

## Supplementary Materials

The following supporting information can be downloaded at: https://www.mdpi.com/article/10.3390/jcm12185825/s1, Table S1: Correlations among parameters in oral diseases patients.
