# Supplementary material for: Role of Macrophage Colony-Stimulating Factor for Staphylococcal Infection in the Oral Cavity
Source: J Clin Med. 2023 Sep 7;12(18):5825. doi: 10.3390/jcm12185825 (PMC10532062; doi:10.3390/jcm12185825)
Supplement: Supplementary file 1 [file jcm-12-05825-s001.zip › jcm-2567048-supplementary.pdf]

Supplemental Table S1 Correlations among parameters in oral diseases patients

	Female		Denture(-)	
	Correlation	*P*-value	Correlation	*P*-value
PPD vs BOP	0.486	0.025*	0.888	0.000**
PPD vs PCR	0.253	0.281	0.504	0.023*
PPD vs TM	-0.080	0.682	0.796	0.000**
BOP vs PCR	-0.061	0.404	0.446	0.049*
BOP vs TM	-0.115	0.612	0.580	0.007**
CAL vs TM	0.545	0.011*	0.829	<0.001**
CAL vs BOP	-0.012	0.949	0.569	0.009**
M-CSF vs Staphylococci	0.448	0.042*	0.490	0.028*
M-CSF vs CA125/MUC16	0.764	0.004**	0.215	0.503

*: $p<0.05$, **: $p<0.01$
